# *Toxoplasma gondii* Reactivation Aggravating Cardiac Function Impairment in Mice

**DOI:** 10.3390/pathogens12081025

**Published:** 2023-08-09

**Authors:** Linding Xie, Yien Xing, Jun Yang, Min Liu, Yihong Cai

**Affiliations:** 1 Department of Health Inspection and Quarantine, School of Public Health, Anhui Medical University, Hefei 230032, China; 2 Department of Microbiology and Parasitology, the Provincial Laboratory of Pathogen Biology of Anhui, and the Key Laboratory of Zoonoses of Anhui, Anhui Medical University, Hefei 230032, China

**Keywords:** *Toxoplasma gondii* Chinese1 genotype Wh6 strain, myocarditis, reactivation, inflammatory infiltrates

## Abstract

Background: *Toxoplasma gondii* (*T. gondii*) reactivation is common, especially among immunocompromised individuals, such as AIDS patients. The cardiac involvement associated with toxoplasmosis, however, is usually obscured by neurological deterioration. The aim of this study was to observe the alterations in cardiac functions in various landmark periods after infection and to assess whether reactivation more seriously damages the heart. Methods: We established three infection models in mice using TgCtwh6, a major strain of *T. gondii* prevalent in China. The groups included an acute group, chronic latent group, and reactivation group. We evaluated the cardiac function impairment via H & E staining, Masson staining, echocardiography, myocardial enzyme profiles, and cardiac troponin, and detected the expression of inflammatory factors and antioxidant factors with Western blotting. Immunofluorescence was used to detect the expression of the macrophage marker F4/80. Results: Our results showed that damage to the heart occurred in the acute and reactivation groups. Impaired cardiac function manifested as a decrease in heart rate and a compensatory increase in left ventricular systolic function. Serum levels of cardiac enzymes also increased dramatically. In the chronic phase, myocardial fibrosis developed, diastolic functions became severely impaired, inflammation persisted, and macrophage expression was slightly reduced. Ultimately, reactivation infection exacerbated damage to cardiac function in mice, potentially leading to diastolic heart failure. Macrophages were strongly activated, and myocardial fibrosis was increased. In addition, the antioxidant capacity of the heart was severely affected by the infection. Conclusions: Taken together, these results suggested that the reactivation of *T. gondii* infection could aggravate injury to the heart, which could be associated with a host-cell-mediated immune response and strong cytokine production by macrophages, thus representing a novel insight into the pathogenic mechanism of toxoplasmosis.

## 1. Introduction

Infections caused by the protozoan parasite *Toxoplasma gondii* (*T. gondii*) occur worldwide, with a high prevalence and potential severity. Clinical manifestations range from asymptomatic to systemic disease [1]. The heart, as the driving force of the body’s circulatory system, can receive direct or indirect effects from a variety of parasites [2]. *T. gondii* tachyzoites can slow their proliferation and become encysted within cardiac myocytes where they become bradyzoites [3,4]. The corresponding trophozoites can be present in macrophages and phagosomes within cardiomyocytes. Therefore, the myocardium is an important site for the storage of *T. gondii* cysts. Toxoplasmosis is the most commonly reported parasitic disease after heart transplantation [5]. The heart is also the second most commonly affected organ after the brain in AIDS patients with toxoplasmosis [6]. The prevalence of *T. gondii* myocarditis in patients with acquired immunodeficiency syndrome exceeds 10% [7], and approximately 12–22% of patients with *T. gondii* infection combined with AIDS present with endomyocardial involvement during autopsy after death [8]. Previous case reports have demonstrated that *T. gondii* infection manifests as myocarditis, pericarditis, arrhythmias, and heart failure in human and animal hearts [9,10,11]. However, this cardiac involvement is often overlooked due to the lack of obvious symptoms or concomitant neurological deterioration, resulting in cardiac effects that have not been widely discussed and explored.

The severity of toxoplasmosis varies depending on individual immune status, genotype, and parasite strain [12]. Chinese 1 (ToxoDB #9) is a genotype isolated from our laboratory that is endemic in Asia, mainly in China [13,14]. The *T. gondii* Chinese1 genotype wh6 strain (TgCtwh6) is one of two representative strains and exhibits similar virulence to the PRU strain (Type II). This strain has a greater propensity to form tissue cysts than other genotypes, leading to latent host infection [15]. Notably, TgCtwh6 continues to parasitize the heart after infection [13].

In this study, we explored the effects of parasite–host interactions on the heart and functional alterations during tachyzoite acute phase proliferation and bradyzoites reactivation by constructing a dexamethasone-induced latent reactivation infection mouse model to mimic an immunosuppressed population. The goal was to determine the pathogenic mechanism of *T. gondii* heart disease in terms of physiology and pathology.

## 2. Materials and Methods

### 2.1. Ethical Approval

This study was conducted according to the guidelines of the Declaration of Helsinki. All experimental procedures were approved by the Biomedical Ethics Committee of Anhui Medical University and performed according to the Guidelines for the Care and Use of Research Animals established by the university (permission no.20211187). All animals were housed in rooms with temperature and humidity set in the appropriate range, along with 12 h dark–light cycles and free access to food and water in standard cages. All efforts were made to minimize the suffering caused to animals in these studies.

### 2.2. Parasites

The Chinese 1 genotype TgCtwh6 strain of *T. gondii* (Toxo DB#9) was used to infect the mice in this study. The TgCtwh6 strain was obtained from the Anhui Provincial Key Laboratory of Microbiology and Parasitology, Anhui Medical University. The cysts of TgCtwh6 were prepared from the brains of female Kunming mice (KM) perorally infected 2 months prior. The brain was homogenized in 3 mL of sterile saline, and the cyst numbers were determined microscopically in 10 μL of suspension [13,16].

### 2.3. Experimental Design

**Figure 1:**
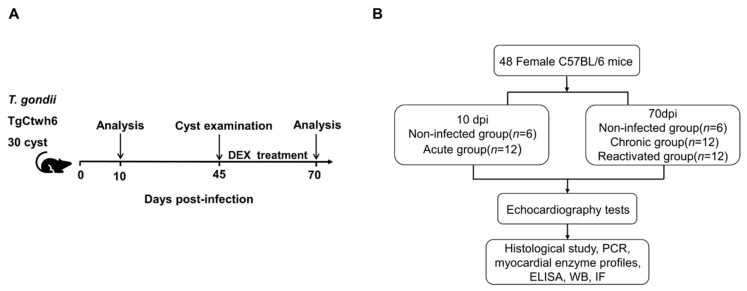
Diagram of the experimental design. (**A**) Diagram of the study stages. (**B**) Experimental protocols. DEX, dexamethasone.

In total, 48 C57BL/6 female mice, 7–8 weeks old, were purchased from Hangzhou Ziyuan Experimental Animal Science and Technology Co., Ltd. (Hangzhou, China) (production license number: SCXK2019-004) and acclimatized for one week for subsequent experiments. Upon arrival, the mice were randomly placed into experimental and control groups according to the experimental protocol, the cages were numbered and sorted for experimental infection, and the mice were analyzed at the designated endpoints (Figure 1A,B). All the infected mice (including the acute group, chronic group, and reactivated group) were infected with 30 cysts of TgCtwh6 from Day 0, and the noninfected (NI) mice were infected with normal saline. To activate dormant *T. gondii*, the glucocorticoid dexamethasone (DEX) was dissolved in the drinking water of the mice at 45 dpi for 25 days at a concentration of 10 mg/L (2.5 mg/kg/day), and the drinking water was changed daily to prevent bacterial cross-contamination [17,18]. The specific groupings of animal were as follows: (i) 10 dpi, acute phase: 6 NI controls and 12 acute infected mice (acute group, AG); (ii) 70 dpi: 6 NI controls, 12 chronically infected mice (chronic group, CG), and (iii) 12 reactivated infected mice (reactivated group, RG). The mice in the reactivated group were treated with DEX between 45 and 70 dpi. A preliminary statistical analysis of the two NI control groups run concurrently with the *T. gondii*-infected groups showed no difference (*p* > 0.05) between the NI controls. For simplicity, where possible, the data collected from all NI mice are pooled and referred to as NI in subsequent graphs and figures.

### 2.4. PCR Detection of the ITS-1 Gene in the Peripheral Blood of T. gondii 

The presence of ITS-1 DNA in the blood means that parasitemia is present and that the latent infection has been successfully activated. DNA was extracted from the peripheral blood of the mice using a DNA extraction kit (AG, Hunan, China) according to the manufacturer’s protocol. PCR amplification of the ITS-1 gene was performed using the primers ITS-1 5′-GATTTGCATTCAAGAAGCGTGATAGTAT-3′ and ITS-1 reverse 5′-AGTTTAGGAAGCAATCTGAAAGCACATC-3′ [19,20]. The PCR mix consisted of 12.5 μL of PCR Master Mix 1× (Promega, WI, USA), 1 μL of each primer at 1 μM, 2.5 μL of 1 × PCR buffer, and 6 μL of DNA sample in a final volume of 25 μL. The reactions were performed in a thermal cycler (Biometra, Gottingen, Germany) with an initial denaturation step of 95 °C for 3 min. This step was followed by 35 cycles, with 1 cycle consisting of 30 s at 55 °C under the annealing temperature and 30 s at 72 °C. DNA loading buffer (AG, Hunan, China) was added to the amplified PCR product, which was then separated via electrophoresis on a 1% agarose gel containing ethidium bromide and recorded using a digital gel documentation system (BioRad, CA, USA). The positive control was the *T. gondii* Chinese 1 genotype wh6 strain.

### 2.5. Determination of Cardiac Function Indicators

A transthoracic echocardiography was performed on the mice before the endpoint of each group of experiments (VINNO 6 VET, Suzhou, China). The mice were anesthetized with isoflurane (RWD, Shenzhen, China) (3% for induction and 1% for maintenance) mixed in 1 L/min 100% O_2_ via a facemask. Parasternal long- and short-axis views were obtained with a high-frequency ultrasound probe. Echocardiographic parameters such as heart rate (HR) were recorded. The left ventricular systolic function of the mice was evaluated using the left ventricular ejection fraction (LVEF) and fractional shortening (LVFS). Cardiac diastolic function was assessed using the mitral ratio of the peak early to late diastolic filling velocity (E/A). The echocardiography operator was blinded to the grouping of the mice.

### 2.6. Histological Analysis

Mouse hearts were fixed with 4% paraformaldehyde universal tissue fixative (Biosharp, Hefei, China), dehydrated with a gradient of ethanol and clear xylene, and then embedded using a biological tissue embedding machine (Yaguan YB-7LF, Hubei, China). Cardiac tissues embedded in paraffin blocks were cut into 4 μm thick sections using a microtome (Leica, Wetzlar, Germany) to examine pathological changes in cardiac tissues via staining with hematoxylin and eosin (H & E) and Masson’s trichrome stain (both from Baso, Zhuhai, China). Images were obtained using a light microscope (Olympus CX41, Tokyo, Japan) equipped with a charge-coupled device camera. 

### 2.7. Biochemical Analysis of Myocardial Enzyme Profiles

Five mice were randomly selected from each group for anesthesia. Orbital blood was collected and centrifuged at 4000× *g* rpm/min for 10 min after 30 min of resting. Each tube of serum was diluted at a specific ratio and tested on the machine. The serum contents, including serum lactate creatine kinase (CK), the creatine kinase-MB fraction (CK-MB), aspartate aminotransferase (AST), dehydrogenase (LDH), and alpha-hydroxybutyrate dehydrogenase(α-HBDH), were determined using a Cobas 8000 automatic biochemistry analyzer (Roche, Basel, Switzerland).

### 2.8. Enzyme-Linked Immunosorbent Assay (ELISA)

The mice were anesthetized with isoflurane, and orbital blood sampling was performed. The blood was left to stand for 30 min, and then serum was taken at 4000 rpm/min for 10 min for the assay. The activity of cardiac troponin I (cTn-I) in the serum was determined using an ELISA kit (Mlbio, Shanghai, China) according to the manufacturer’s protocol. The concentrations of these analytes were calculated from the absorbance values at 450 nm using an enzyme marker (Infinite F50, Tecan, Männedorf, Switzerland). The concentrations were calculated from the standard curves using Microsoft Excel.

### 2.9. Western Blot Analysis

Protein extracts from the whole heart were separated using SDS-polyacrylamide gels and then transferred onto nitrocellulose membranes. Rabbit polyclonal antibodies against IFN-γ, COX2 (both from Wanleibio, Shenyang, China), TNF-α (Affinity Bioscience, OH, USA), and SOD1 (Proteintech, Wuhan, China) and mouse polyclonal antibodies against *Tg*BAG1 (laboratory homemade) were incubated with the membranes overnight at 4 °C. The protein levels were normalized to those of GAPDH (Proteintech, Wuhan, China). The membranes were washed in TBST (20 mM Tris-HCl, pH 7.5, 150 mM NaCl, 0.1% Tween 20) 5 times, incubated with the indicated HRP-conjugated goat anti-rabbit/mouse IgG (both from Proteintech, Wuhan, China) antibody for 1 h at room temperature, and then washed in TBST 5 times. The protein bands were observed using an enhanced chemiluminescence detection system (BioRad, CA, USA) and photographed.

### 2.10. Immunofluorescence Assay

Immunofluorescence (IF) staining was performed to identify the infiltrated macrophages in the mouse myocardium by incubating the tissues with mouse anti-F4/80 (1:100, Biolegend, CA, USA) at 4 °C overnight. The nuclei of the cells were stained with a mounting medium (antifading, with DAPI, Solarbio, Beijing, China). Finally, the cells were observed and photographed with a Leica DM6 B Orth fluorescence microscope.

### 2.11. Statistical Analysis

GraphPad Prism 8.0.2 software was used for the statistical analysis and graphing. Survival data were analyzed via the Kaplan–Meier method and compared using a log-rank test. A two-tailed Student’s *t*-test was applied to determine the statistical significance between the two groups. ANOVA tests were applied to the multiple sets of data. Statistical differences were considered significant when *p* < 0.05. All the graphic data are presented as the mean ± SD values.

## 3. Results

### 3.1. Establishment of Dexamethasone-Induced Recurrence in TgCtwh6-Infected Mice

**Figure 2:**
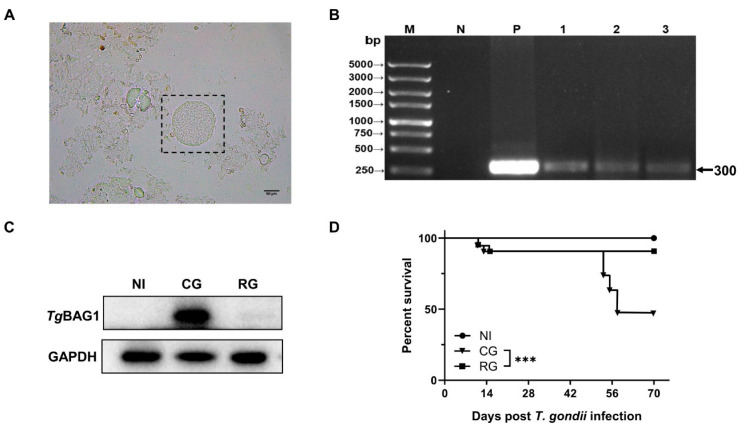
Modeling of latent and reactivated *T. gondii* infection. (**A**) TgCtwh6 homogenized smear of brain tissue from chronically infected mice, ×400, Scale bar = 50 μm. (**B**) PCR results of *T. gondii* ITS-1 in peripheral blood of mice; M, DNA marker; N and P represent negative and positive controls; lanes 1–3 are the PCR results of peripheral blood from reactivated mice (*n* = 3). (**C**) Western blot analysis showed that *Tg*BAG1 was increased in chronically infected hearts and decreased after reactivation, GAPDH was used as an internal control. (**D**) The survival rates of the mice after TgCtwh6 infection/and DEX treatment (*n* = 12). NI, noninfected; AG, acute group; CG, chronic group; RG, reactivated group; *** *p* < 0.001.

The mice entered the acute phase at 9 dpi. At this stage, they developed weight loss, a mild loss of appetite, ruffled hair, photophobia, and white discharge from their eyes. The chronic phase was entered on Day 45 after infection, and TgCtwh6 cysts were confirmed in the mouse brain tissue homogenates (Figure 2A). *Tg*BAG1, which is only expressed in bradyzoites, is a specific and characterized protein with a bradyzoite form which acts as a marker for *T. gondii* cyst infections [21]. During this period, *Tg*BAG1 was highly expressed in the heart tissue, ITS-1 was negative in the blood, and the parasitemia disorder disappeared (Figure 2B,C). DEX was used to stimulate the incubation period and model *T. gondii* activation. After 25 days of DEX administration, there was a significant decrease in *Tg*BAG1 in heart tissue and positive ITS-1 in the blood (Figure 2B,C). TgCtwh6 was then activated in the mice, and the survival rate decreased to 50% (Figure 2D). 

### 3.2. Reactivation Could Aggravate Pathological Damage in the Hearts of Mice

**Figure 3:**
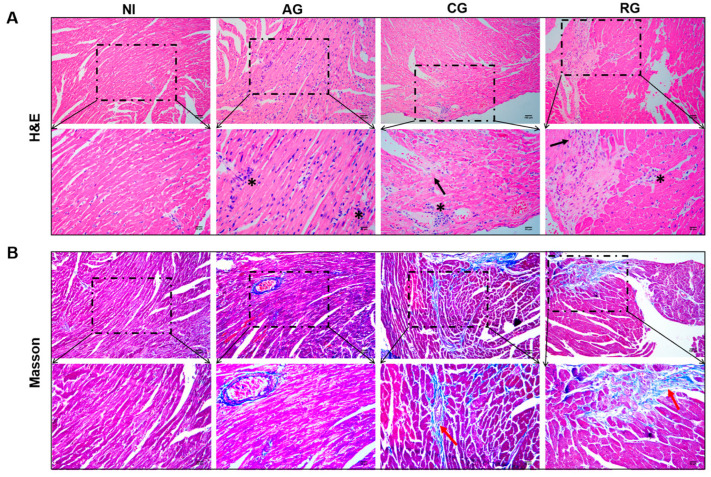
Histopathology changes in the hearts of mice at various periods of *T. gondii* infection and post-infection. (**A**) H & E staining; magnification, 200× and 400×, scale bar of the upper images = 100 µm, scale bar of the lower images = 50 µm. (**B**) Masson staining; magnification, 200× and 400×, scale bar of the upper images = 100 µm, scale bar of the lower images = 50 µm. Inflammatory infiltration (asterisk), myocardial fiber sparing (black arrows), and collagen fiber deposition (red arrows). NI, noninfected; AG, acute group; CG, chronic group; RG, reactivated group.

To investigate the effects of different post-infection times on the hearts of the hosts and observe pathological changes in the myocardial structure, H & E staining and Masson staining were performed (Figure 3A,B). In the acute group, the cardiac tissue showed a large diffuse/local inflammatory infiltrate (asterisks), and no significant fibrotic features were observed. The myocardial fibers in the chronic group were disorganized and loose (black arrows). The myocardial cell structure was impaired with focal inflammatory cell infiltration (asterisks). Masson staining showed collagen fiber deposition (red arrows) in the myocardium; this deposition was significantly greater than that in the NI group. Myocardial fibers in the reactivated group were disorganized (black arrows) and accompanied by focal inflammatory cell infiltration (asterisks). In the left pale region, the myocardium was damaged, and tissue scarring was visible. In addition, myocardial interstitial fibrosis persisted (red arrows).

### 3.3. Effects of TgCtwh6 Infection on the Heart Functions of Mice at Various Periods after Infection

**Figure 4:**
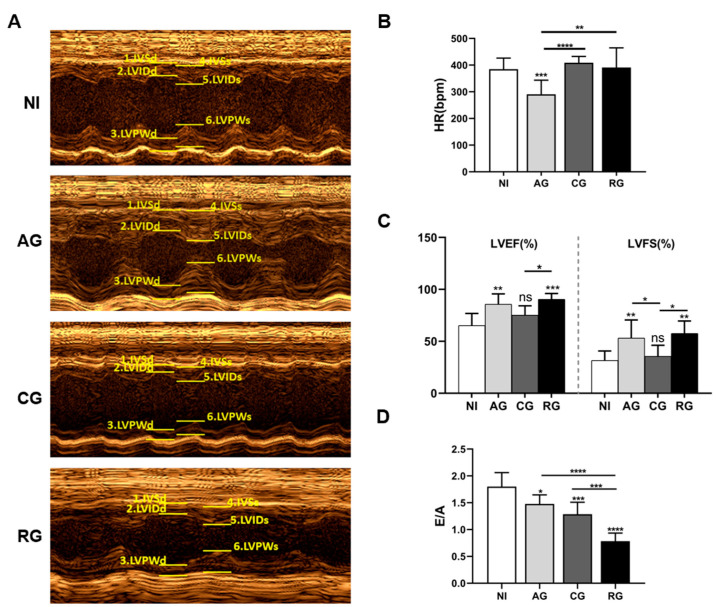
Heart functions of mice during various periods of *T. gondii* infection and post-infection. (**A**) Representative M-mode echocardiograph. (**B**) HR; (**C**) LVEF and LVFS. (**D**) E/A were determined by echocardiography. Data are presented as the mean ± SD (*n* = 6–9). NI, noninfected; AG, acute group; CG, chronic group; RG, reactivated group. IVSd, interventricular septum thickness in diastole; LVIDd, left ventricular internal diameter at end diastole; LVPWd, left ventricular posterior wall thickness in diastole; IVSs, interventricular septal thickness at end systole; LVIDs, left ventricular internal dimension in systole; LVPWs, left ventricular posterior wall end systole, LVEF, left ventricular ejection fraction; LVFS, left ventricular fractional shortening; HR, heart rate. * *p* < 0.05, ** *p* < 0.01, *** *p* < 0.001, **** *p* < 0.0001, ns, not significant.

To observe changes in the thickness of the anterior and posterior walls of the hearts along with the changes in the cardiac chambers, we used echocardiography to visualize the function and structure of the hearts in each group of mice (Figure 4A). There was a significant decrease in the heart rates of the mice in the acute phase and no significant changes in the other groups (Figure 4B). Cardiac alterations were evaluated in terms of changes to both systolic and diastolic functions. Notably, LVEF and LVFS underwent compensatory elevation during the acute and activated phases (Figure 4C). This result indicates that TgCtwh6 increased the host cardiac load during the tachyzoite phase. Compared to the control group, E/A decreased successively starting from the acute and chronic phases (Figure 4D). The significant decrease in mitral flow E/A < 1 in the reactivated group indicates that severe diastolic heart failure may have occurred at this time (Figure 4D). 

### 3.4. Reactivation of T. gondii Increased the Various Cardiac Enzymes and Cardiac Troponin in Mice

**Figure 5:**
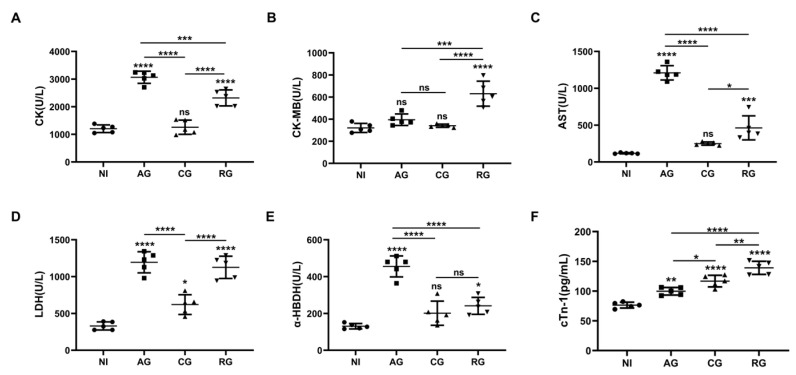
*T. gondii* infection causes alterations in myocardial enzyme profiles and cardiac troponin. (**A**) Myocardial zymograms containing CK, (**B**) CK-MB, (**C**) AST, (**D**) LDH, and (**E**) α-HBDH in mouse serum created using an automatic biochemistry analyzer (*n* = 5). (**F**) cTn-I in mouse serum was determined through an ELISA. The data show the values ± SEM of the mice. NI, noninfected; AG, acute group; CG, chronic group; RG, reactivated group; CK, creatine kinase; CK-MB, creatine kinase-MB fraction; AST, aspartate aminotransferase; LDH, lactate dehydrogenase; α-HBDH, alpha-hydroxybutyrate dehydrogenase. * *p* < 0.05, ** *p* < 0.01, *** *p* < 0.001, **** *p* < 0.0001, ns, not significant.

As myocardial injury and lesions may lead to changes in various biochemical parameters such as cardiac enzymes, we further analyzed the serum levels of CK, CK-MB, AST, LDH α-HBDH, and cTn-I in the mice. The results showed that the levels of CK, AST, LDH, α-HBDH, and cTn-1 were significantly higher in the acute phase compared with the normal mice and gradually returned to normal levels in the chronic phase (Figure 5A,C–F). However, the levels of LDH and cardiac troponin cTn-1 remained upregulated in the chronic phase compared to the controls (Figure 5D,F). After DEX activation, all indicators were significantly upregulated, indicating that reactivation had a significant effect on the damage to the host heart. In contrast to the other indicators, CK-MB levels underwent a significant increase only during the reactivated phase (Figure 5B). cTn-1 levels continued to increase with the duration of infection (Figure 5F). 

### 3.5. Alterations in Antioxidant and Inflammatory Effects in the Heart during Various Periods after Infection

**Figure 6:**
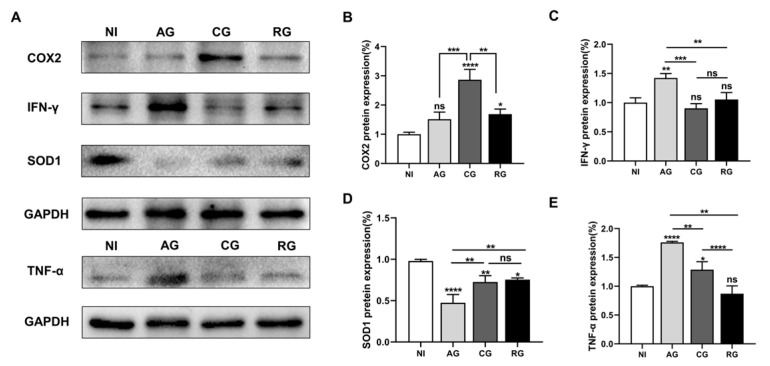
*T. gondii* infection affects antioxidant and inflammatory activities. (**A**) Western blot analysis showing the protein levels. (**B**) COX2, (**C**) IFN-γ, (**D**) TNF-α, and (**E**) SOD1; GAPDH was used as an internal control; NI, noninfected; AG, acute group; CG, chronic group; RG, reactivated group; * *p* < 0.05, ** *p* < 0.01, *** *p* < 0.001, **** *p* < 0.0001, ns, not significant.

Excessive inflammation plays a key role in the progression of parasite-induced myocardial injury. Therefore, in the present study, we first investigated the levels of pro-inflammatory cytokines and oxidative stress factors in the heart tissues of each group of mice (Figure 6A). Our analysis showed that COX2 protein levels were significantly higher in the chronic phase and decreased in the activation phase (Figure 6B). Compared to the controls, IFN-γ and TNF-α in the cardiac tissue were higher in the acute group and decreased in the chronic and reactivation phases (Figure 6C,E). Interestingly, the expression of SOD1 protein in the heart significantly decreased after parasite infection and increased after the chronic phase (Figure 6D). Taken together, these results suggest that *T. gondii* infection causes inflammation and affects antioxidant capacity.

### 3.6. Effect of T. gondii Infection on Macrophage Infiltration In Vivo

**Figure 7:**
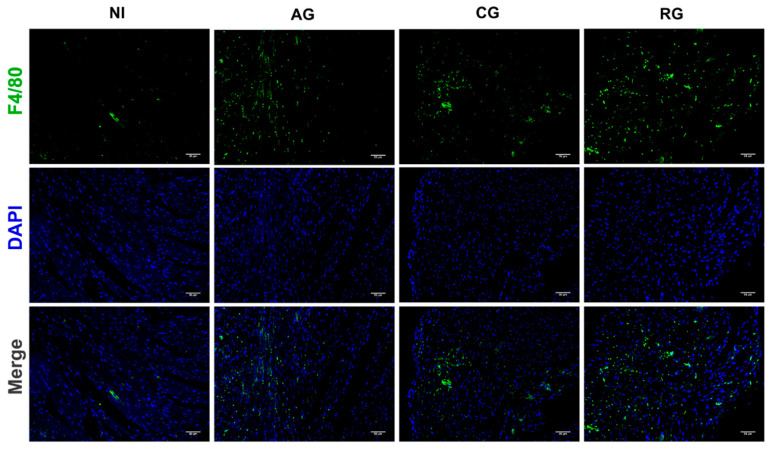
Macrophage infiltration in the hearts of mice at different periods of *T. gondii* infection and after infection. Immunofluorescent images indicating F4/80 (green) in the heart at 200× with DAPI (blue), Scale bar = 50μm. NI, noninfected; AG, acute group; CG, chronic group; RG, reactivated group.

Given that excessive macrophage activation is responsible for the production of proinflammatory mediators and the promotion of inflammation-induced cardiac lesions during parasitic infection, we further analyzed the percentage of infiltrating macrophages in the heart tissue of each group of infected mice (Figure 7). The results of the IF staining showed that invasion of TgCtwh6 activated the expression of infiltrated F4/80 macrophages in the myocardium of mice. The percentage of infiltrated F4/80 macrophages in the myocardium of latently infected mice was slightly but significantly lower than that in the acute group. However, with the activation of *T. gondii* in the heart, the infiltration of inflammation-associated macrophages was aggravated to levels even higher than those in the acute phase. 

## 4. Discussion

The interactions between *T. gondii* and cardiomyocytes are largely unknown, and it remains unclear how *T. gondii* affects the function of the host heart. DEX successfully induced *T. gondii* recurrence in persistently infected mice, mimicking the effects observed in immunocompromised human populations such as AIDS patients [18,22]. In this study, three period-based experimental groups were established to determine the effects of *T. gondii* on the host myocardium at different stages. Since oral infection is the main form of *T. gondii* infection, we analyzed this vector in the present study. After ingestion, *T. gondii* rapidly divided into the intestinal epithelium and spread to the brain, heart, and other distant organs via lymphatic vessels and bloodstream invasion [23,24,25]. It was shown that *T. gondii* trophozoites can be found in the phagosomes within macrophages and cardiomyocytes [26]. The parasites are driven by an action-myosin-dependent gliding motility mechanism, establishing intracellular vesicles [27]. It is likely that this remodeling prevents lysosomal fusion, leading to intracellular survival of the parasite [28].

CK, CK-MB, LDH, α-HBDH, and AST are among the primary component enzymes of the myocardial enzyme profile and are present at high levels in myocardial tissue [29]. When myocardial tissue is attacked by parasites, cell membrane integrity is disrupted, permeability increases, and enzymes in myocardial cells are slowly released and subsequently enter the blood, causing a significant increase in the activity of these enzymes in the blood [30]. In the present study, TgCtwh6 tachyzoites induced inflammatory cytokines with oxidative stress. This process caused damage to cardiomyocytes, resulting in a large release of CK, LDH, and AST into the blood and leading to increased concentrations. Our results suggest that *T. gondii*’s damage to the host myocardium is dynamic. In addition, the effects of *T. gondii* on cardiac function after activation were significant. CTn-1 is a structural protein of the myocardium and has a very high sensitivity and specificity for diagnosing myocardial injury and necrosis [31]. Our study showed that the serum levels of this protein in the myocardium increase over time. This result suggests that the longer a *T. gondii* infection persists, the more severe the damage to the host heart will be, especially in immunocompromised patients. In addition, cTn-1 plays an important role in myocardial contraction [32]. When evaluating cardiac function, we found that both LVEF and LVFS were compensated by an increase in the acute and activation phases, with a gradual decrease in E/A and an activation phase with E/A < 1. Our results suggest that TgCtwh6 infections mainly affect the diastolic function of the heart, defined as diastolic heart failure, also known as ejection fraction preserved heart failure. The reactivation of latent *T. gondii* increases the burden on the host heart and leads to severe impairment of cardiac function. However, to date, no conclusively effective treatment has been identified for this disease [33].

The presence of tachyzoites in blood or lymphatic fluid is the key differential factor in diagnosing the acute phase of *T. gondii* infection [34]. Intense parasitemia induces a greater response from the host immune system [35]. Th1 overreaction and intense inflammatory responses can lead to host pathological changes that cause damage to host tissues. Studies have shown that host control of *T. gondii* is largely dependent on the cytokine IFN-γ, which activates iNOS to produce NOx radicals that directly kill intracellular bacteria and protozoa [36]. TNF-α is the earliest and most important inflammatory mediator that appears during the inflammatory response. TNF-α activates neutrophils and lymphocytes, increases vascular endothelial cell permeability, and regulates other tissues’ metabolic activities. This mediator also drives other synthesis processes and releases other cytokines [37]. Importantly, TNF-α and IFN-γ are important cytokines produced after *T. gondii* infection and are essential for controlling tachyzoite replication during acute infection [38]. In our study, TNF-α and IFN-γ expression was significantly increased during the acute phase of TgCtwh6 infection and decreased during the chronic phase. After activation, IFN-γ expression was elevated again but appeared to be inhibited by DEX. DEX has immunosuppressive and anti-inflammatory effects [39]. In agreement with these results, in our study, DEX intake slightly reduced the severity of inflammation but led to an increase in myocardial necrosis. The mediation of inflammation through immune cells is another key factor. This activity both exacerbates the damage caused by the aggressor and regulates the repair of cardiac structures [40]. Therefore, it is crucial to strictly regulate host immune responses to *T. gondii* infection.

The development and regression of myocardial fibrosis due to parasitemia is caused by a specific host–parasite relationship characterized by a cell-mediated immune response to the host, followed by intense cytokine production by macrophages [41]. Resident macrophage populations recognize danger signals from cardiomyocytes. Then, these populations recruit immune cells and produce harmful inflammation to clear the infection. Our study found that the macrophage marker F4/80 was highly responsive when the host was attacked by tachyzoites in the myocardium during the acute and reactivation periods. At the same time, expression of the pro-inflammatory factor IFN-γ was similarly elevated. These results suggest that cardiac immune cells are activated by microorganisms that recruit different populations of leukocytes into the inflamed heart tissue. During the initial inflammatory phase, phagocytosis and the clearance of dead cells was achieved through the expansion of neutrophil and macrophage populations [42].

In addition, macrophages in the heart have a powerful repair role in cardiac inflammation, fibrosis, and tissue [43]. An analysis of the Masson staining showed significant differences in collagen deposition within the chronic and activated groups compared to that of the control and acute groups. The induction of inflammatory factor COX2 in failing hearts is associated with the presence of myocardial scarring (inflammation and fibrosis) [44], which is consistent with our results. Overall, reactivation significantly aggravates myocardial fibrosis and myocardial injury in mice. Myocardial tissue has a complex and organized collagen network, mainly type I and III, and plays a key role in the regulation of apoptosis in pathological deformation, cardiac expansibility, and force transmission [45]. Thus, maintaining this tissue is essential for cardiac function. Cardiac fibrosis is the inevitable result of long-term damage to the heart muscle, which can lead to wall thickening, impairment of systolic and diastolic functions, and even a decrease in overall heart function [46]. The healing process is initiated via the activation of myofibroblast proliferation and injury to myocardial neovascularization [47]. This long-term latent myocardial damage caused by *T. gondii* eventually leads to inevitable myocardial fibrosis. However, uncontrolled tissue damage and the continuous activation of pro-inflammatory signals eventually lead to impaired cardiac function and contribute to heart failure.

Moreover, oxidative stress underlies many inflammatory disease mechanisms, and inflammation is also involved in the induction of oxidative stress [48]. In this study, the protein expression of SOD1 (a cytoplasmic Cu-Zn-SOD) was significantly decreased after *T. gondii* infection, indicating oxidative stress in *T. gondii* hosts. *T. gondii* infection also affected the redistribution of metal ions in the host. The transporter protein ZIP8 of Zn was shown to be protective towards *T. gondii*-induced acute hepatocyte injury in mice [49]. Surprisingly, SOD1 was again elevated in the chronic phase and reactivation period. This result suggests that Cu and Zn may be involved in the pathogenesis of *T. gondii*. The exact mechanism, however, remains to be determined.

## 5. Conclusions

*T. gondii* infection leads to the development of myocarditis and oxidative stress. The reactivation of *T. gondii* infection could aggravate injury to the heart, which might be associated with a host-cell-mediated immune response and strong cytokine production by macrophages. *T. gondii*’s damage to the heart was significant, and tachyzoites attacked more intensely than bradyzoites. These observations may be crucial to understanding how myocardial damage leads to reactivation of *T. gondii* infections. The results of the present study provide an experimental basis for establishing the prevention and treatment of toxoplasmosis.

## Data Availability

Not applicable.
